# P-1444. Impacts of LGBT Equality Index and 2016 UN Resolution Vote on AIDS-Related Mortality

**DOI:** 10.1093/ofid/ofae631.1616

**Published:** 2025-01-29

**Authors:** Neil Modi, Kevin Ikuta

**Affiliations:** UCLA, Los Angeles, California; West Los Angeles VA, Los Angeles, California

## Abstract

**Background:**

Stigma surrounding HIV has been well-described as a barrier to HIV care engagement. Much of this arises from a belief that only certain groups of people can get HIV as well as public opinion and government policies toward LGBT people. Recognizing the effects of discriminatory laws against individuals based on sexual orientation and gender identity, the United Nations Human Rights Council adopted resolution A/HRC/RES/32/2 in 2016, deploring acts of violence and discrimination based on these traits. Our study had two aims: first, to assess if a country’s AIDS-related mortality differed based on how the country voted on the 2016 resolution and, second, to describe whether greater legal protection of LGBT rights was associated with reduced AIDS-related mortality.

**Figure:**
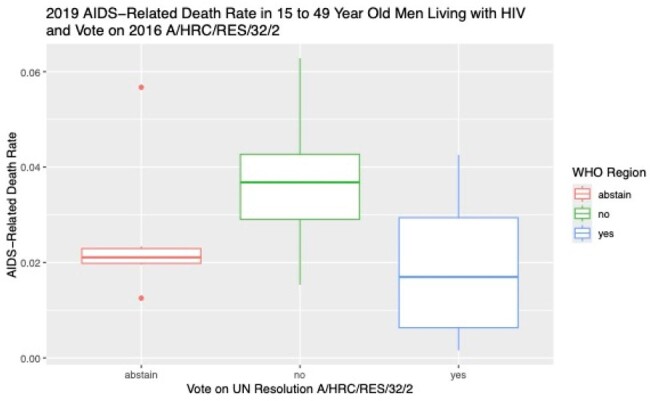

**Methods:**

We used HIV prevalence and mortality estimates in men aged 15-49 from the Global Burden of Disease 2019 to determine the case fatality rate for men aged 15-49 living with HIV by country. To estimate the legal protection of LGBT rights, we utilized the Equaldex Equality Index, a publicly available rating from 0 to 100 describing LGBT legal protections and public opinion by country, with higher values reflecting greater equality. We then performed multivariate log-linear regression to describe the relationship between a country’s AIDS-related mortality among men 15-49 and that country’s Equality Index and vote on the 2016 resolution, adjusted for WHO region.

**Figure:**
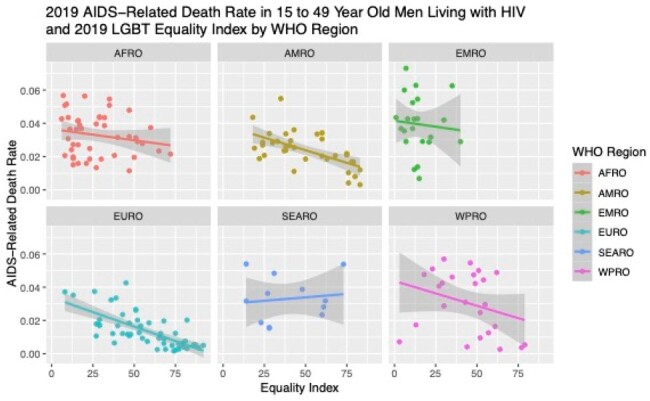

**Results:**

AIDS-related fatality rate was available for 204 countries/territories, an Equality Index was available for 196 countries, and 47 countries voted. In the countries that voted in favor, there was a 65% (95% CI 43-79%, p< .001) relative risk reduction in AIDS-related fatality rate. When adjusted for WHO region, the relative risk reduction was 72% (95% CI 35-88%, p< .06). Assessing the impact of the Equality Index, multivariate log-linear analysis adjusting for region showed that for every 1 point increase there was a 1.6% (CI 1.2-2.0%, p< 0.001) relative risk reduction in AIDS-related fatality rate.

**Conclusion:**

The results of this study support the hypothesis that increasing LGBT legal protection can improve HIV care engagement. It also highlights the significant impact public opinion and government policies toward LGBT people has on AIDS-related mortality.

**Disclosures:**

**All Authors**: No reported disclosures

